# Supplementary material for: Sp1-regulated expression of p11 contributes to motor neuron degeneration by membrane insertion of TASK1
Source: Nat Commun. 2019 Aug 22;10:3784. doi: 10.1038/s41467-019-11637-4 (PMC6706379; doi:10.1038/s41467-019-11637-4)
Supplement: Supplementary file 1 — Supplementary Information [file 41467_2019_11637_MOESM1_ESM.pdf]

Supplementary information

Sp1-regulated expression of p11 contributes to motor neuron degeneration by membrane insertion of TASK1

García-Morales et al.

SUPPLEMENTARY NOTE 1

siRNA$_{p11}$-induced hyperpolarization in SMNs^{wt} (Fig. 1l) could affect Ca^{2+} influx via GluRs and VSCCs. To test this hypothesis, selective inhibitors for NMDA (APV) and AMPA (NBQX) receptors, as well as for L-type (nifedipine), N-type (ω-conotoxin) or P/Q-type (ω-agatoxin) VSCCs were added to the bath solution before and during the excitotoxic challenge (Supplementary Fig. 3g). siRNA$_{p11}$ strongly attenuated the drop of resting $[Ca^{2+}]_i$ induced by APV, nifedipine, and ω-conotoxin, but not by NBQX and ω-agatoxin (Supplementary Fig. 2h,i). These results suggest that p11 knockdown reduced Ca^{2+} entry through NMDA receptors, as well as L-type and N-type channels, but not via AMPA receptors or P/Q-type channels, at baseline. Interestingly, while APV reduced peak $[Ca^{2+}]_i$ (-33.4 ± 3.1%), NBQX fully blocked the rapid response to glutamate in cRNA-treated SMNs^{wt}. In addition, siRNA$_{p11}$-induced reduction in peak $[Ca^{2+}]_i$ was accentuated by APV, but unchanged by any of the VSCCs inhibitors (Supplementary Fig. 3j). These findings agree with the relief of Mg^{2+} block of NMDA receptors induced by Vm depolarization and/or AMPA receptors-mediated fast excitation[1,2].

Strikingly, APV fully prevented glutamate-induced Ca^{2+} deregulation in cRNA-treated SMNs^{wt}, whereas AMPA receptors and P/Q-type VSCCs inhibitors drastically decreased the percentage of them undergoing deregulation (Supplementary Fig. 3f). siRNA$_{p11}$ strengthened the effects of AMPA, L-, N-, and P/Q-type inhibitors since the fraction of SMNs^{wt} suffering Ca^{2+} deregulation was reduced in the presence of NBQX, nifedipine, ω-conotoxin, and ω-agatoxin (Supplementary Fig. 3f). Within the analyzed time window, contribution of L- and N-type channels to glutamate-induced Ca^{2+} deregulation was lesser than that of P/Q-type. The strengthening of L-/N-type dependence under siRNA$_{p11}$ treatment could be the result of two mechanisms. First, p11 down-regulation induces Vm

hyperpolarization, thereby reducing opening probability of non-fully inhibited L- and/or N-type channels under the drug concentrations used in our experiments. Second, if full inhibition of L- and/or N-type channels had been reached in our experiments, siRNA$_{p11}$-induced hyperpolarization would be expected to reduce Ca^{2+} entry via NMDARs and/or non-targeted VSCCs. Furthermore, in the few cells than experienced Ca^{2+} deregulation, the L-type, but not the N-type, inhibitor prevented siRNA$_{p11}$-promoted delays in Ca^{2+} deregulation (Supplementary Fig. 3k). These results suggest that, at least in the fraction of cells that still was glutamate-sensitive in the time window we studied, siRNA$_{p11}$-induced delay in Ca^{2+} deregulation was L-type-dependent. None of the siRNA$_{p11}$-treated SMNs^{wt} showed this last phase under APV, NBQX, and ω-agatoxin (Supplementary Fig. 3f). Therefore, p11 downregulation attenuates Ca^{2+} entry into the motoneuron through GluRs and VSCCs.

SUPPLEMENTARY FIGURES

Supplementary Figure 1

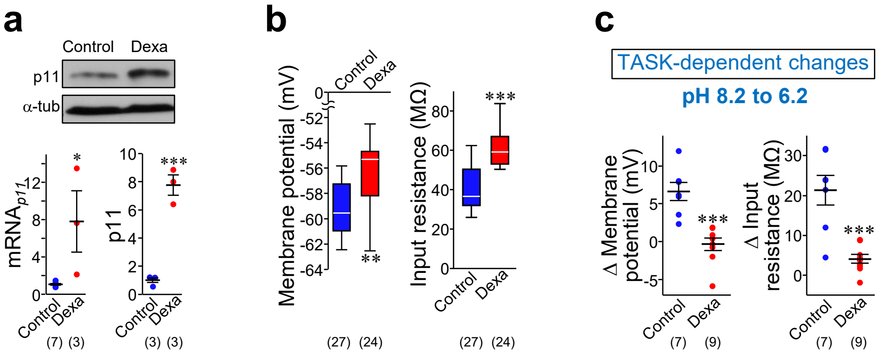

Supplementary Figure 1. Dexamethasone upregulates p11, increases motoneuron IME, and reduces TASK-dependent changes. Chronic incubation (≥ 4 h) of brainstem slices with dexamethasone (Dexa., 1 μM) increased mRNA$_{p11}$/p11 levels in the HN (**a**), enhanced IME (**b**), and reduced the impact of pH changes on Vm, and R_N (**c**) of HMNs. *gapdh* and α-tub were the housekeeping gene for *q*RT-PCR and the internal protein loading reference for western blotting, respectively. Number of independent samples is in parentheses. Summary data are shown as mean ± s.e.m. **p* < 0.05, ***p* < 0.01, ****p* < 0.001; n.s., not significant; by Mann-Whitney *U* test (**a**) or Student *t*-test (**b**,**c**). Source data are provided as a Source Data file.

Supplementary Figure 2

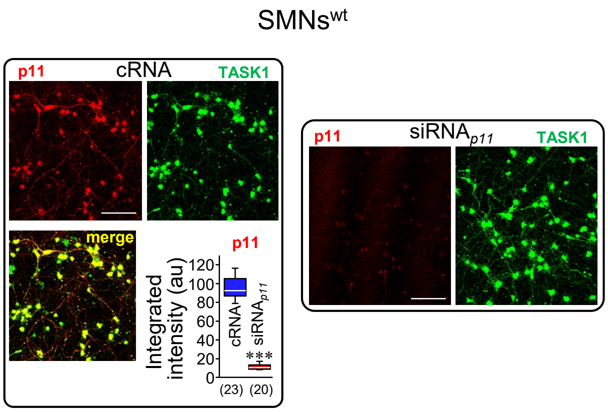

Supplementary Figure 2. siRNA$_{p11}$ efficiently reduced p11 immunolabelling in SMNs^{wt}. Effect of siRNA$_{p11}$ or cRNA (2 μM) on p11 and TASK1 expression determined in SMNs^{wt} by immunofluorescence analysis. Experimental protocol as in figure 1i. Box-plot of the integrated intensity (in arbitrary units, au) of p11 immunofluorescence after the indicated treatments ($n \geq 6$ fields for each of 3 independent experiments). Number of independent samples is in parentheses. Scale bars, 100 μm. ***$p < 0.001$; by Student t-test. Source data are provided as a Source Data file.

Supplementary Figure 3

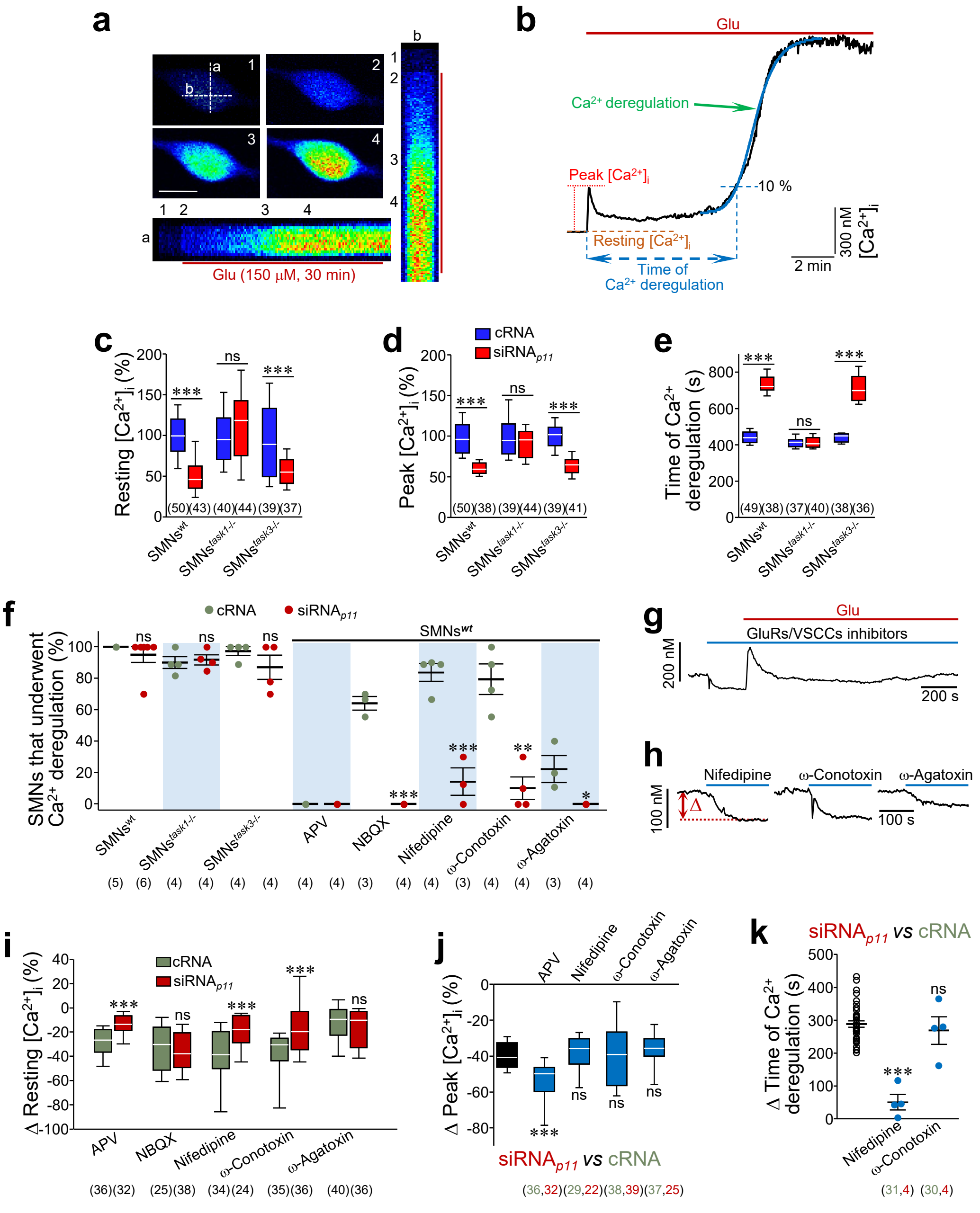

Supplementary Figure 3. siRNA$_{p11}$ attenuates Ca^{2+} influx through GluRs and VSCCs in motoneurons via TASK1. (**a,b**) Confocal images (**a**) and time course of Ca^{2+} dynamic (**b**) from two Fluo-4 charged SMNs^{wt} indicating resting (1), Glu-induced early rapid peak (2), Ca^{2+} deregulation (3) and subsequent sustained and irreversible plateau (4) phases. a and b, time-lapse (0.2 Hz) of relative fluorescence, at lines of interest indicated in panel 1, during the complete time course of recording. Time of Ca^{2+} deregulation was the interval between Glu-induced rapid response and 10% raise of the fitted sigmoidal curve to Ca^{2+} deregulation interval (blue line). Scale bar: 20 µm. (**c-e**) Box-plots of indicated parameters, for siRNA$_{p11}$- and cRNA-treated SMNs. The mean value for each cRNA-treated pool was taken as 100%. Cells are the same as in figure 2a,b. (**f**) Percent of total number of SMNs that experienced Ca^{2+} deregulation during Glu exposure for each genotype and treatment. (**g**) Representative time course of $[Ca^{2+}]_i$ alterations illustrating the experimental protocol to study the impact of GluRs/VSCCs inhibitors (blue line) on Ca^{2+} dynamics. Red lines indicate Glu exposure interval. (**h-k**) Representative recordings (**h**) and averaged changes induced in resting $[Ca^{2+}]_i$ (**i**), peak $[Ca^{2+}]_i$ (**j**), and time of Ca^{2+} deregulation (**k**) by addition to the bath of the indicated GluRs/VSCCs inhibitors. In **j** and **k**, untreated conditions cells were the same than in **d** and **e**; changes in siRNA$_{p11}$ relative to cRNA condition are represented. Number of independent samples is in parentheses. Summary data are shown as mean ± s.e.m. *$p < 0.05$, **$p < 0.01$, ***$p < 0.001$; n.s., not significant; by Student *t*-test (**c-e,i-k**) or Mann-Whitney test (**f**). Source data are provided as a Source Data file.

Supplementary Figure 4

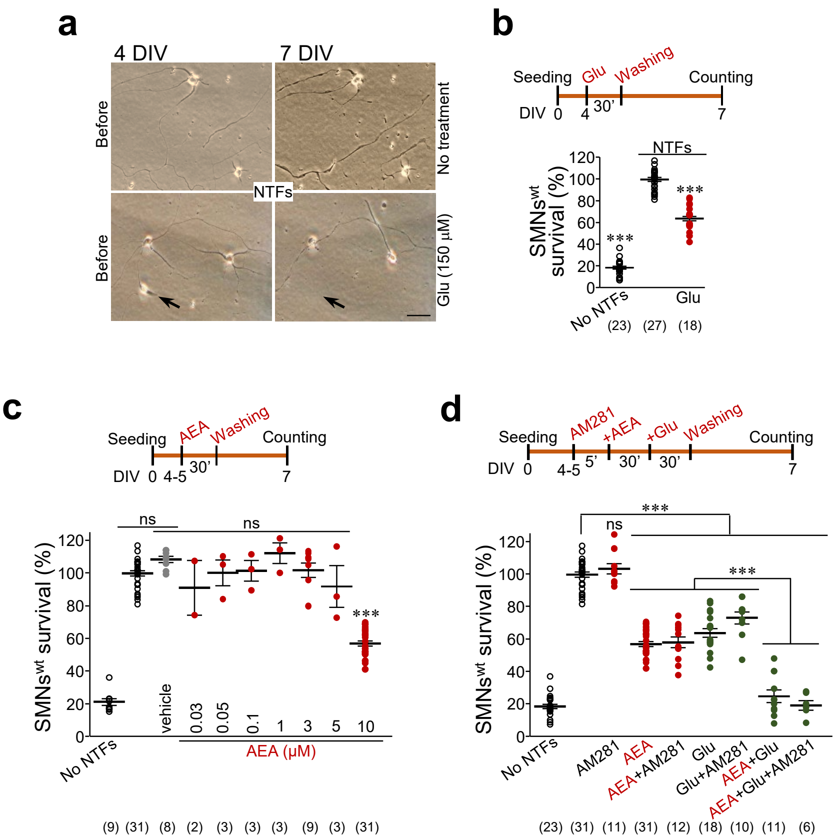

Supplementary Figure 4. Vulnerability of SMNs^{wt} to glutamate and the endocannabinoid anandamide (AEA). (**a**,**b**) Photomicrographs (**a**) of two wells from a SMNs^{wt} culture at 4 and 7 DIV, untreated (top) or subjected to an excitotoxic stimulus (bottom; Glu, 150 µM, 30 min) at 4 DIV (see schematic in **b**, top). Arrows indicate absence of a SMN^{wt} after Glu-treatment. Scale bar: 50 µm. Plot (**b**) showing effects of Glu on SMNs^{wt} survival at 7 DIV. Culture medium was always supplemented with neurotrophic factors (NTFs). Incubation with culture medium without NTFs (No NTFs), from the day of SMNs seeding, was taken as minimal survival expected in our cultures. (**c**) Dose-dependent effects of AEA, added at the indicated concentrations (ranging from 0.03 to 10 µM), on SMNs^{wt} survival following the experimental protocol schematized on top. In the vehicle condition the same volume of solution in which AEA was solved, ethanol (0.07%, v/v), was added to the culture medium for 30 min. (**d**) Histogram showing the effects of AEA (10 µM, 60 min), AM281 (0.5 µM, 65 min) or Glu (150 µM, 30 min) alone, or at the indicated combinations, on SMNs^{wt} survival. Schematic of the experimental protocol is represented on top. In all survival experiments, the mean value for untreated NFTs condition was taken as 100%. Number of independent samples is in parentheses. Summary data are shown as mean ± s.e.m. ***$p < 0.001$; n.s., not significant; by one-way ANOVA with *post hoc* Holm-Sidak method. Source data are provided as a Source Data file.

Supplementary Figure 5

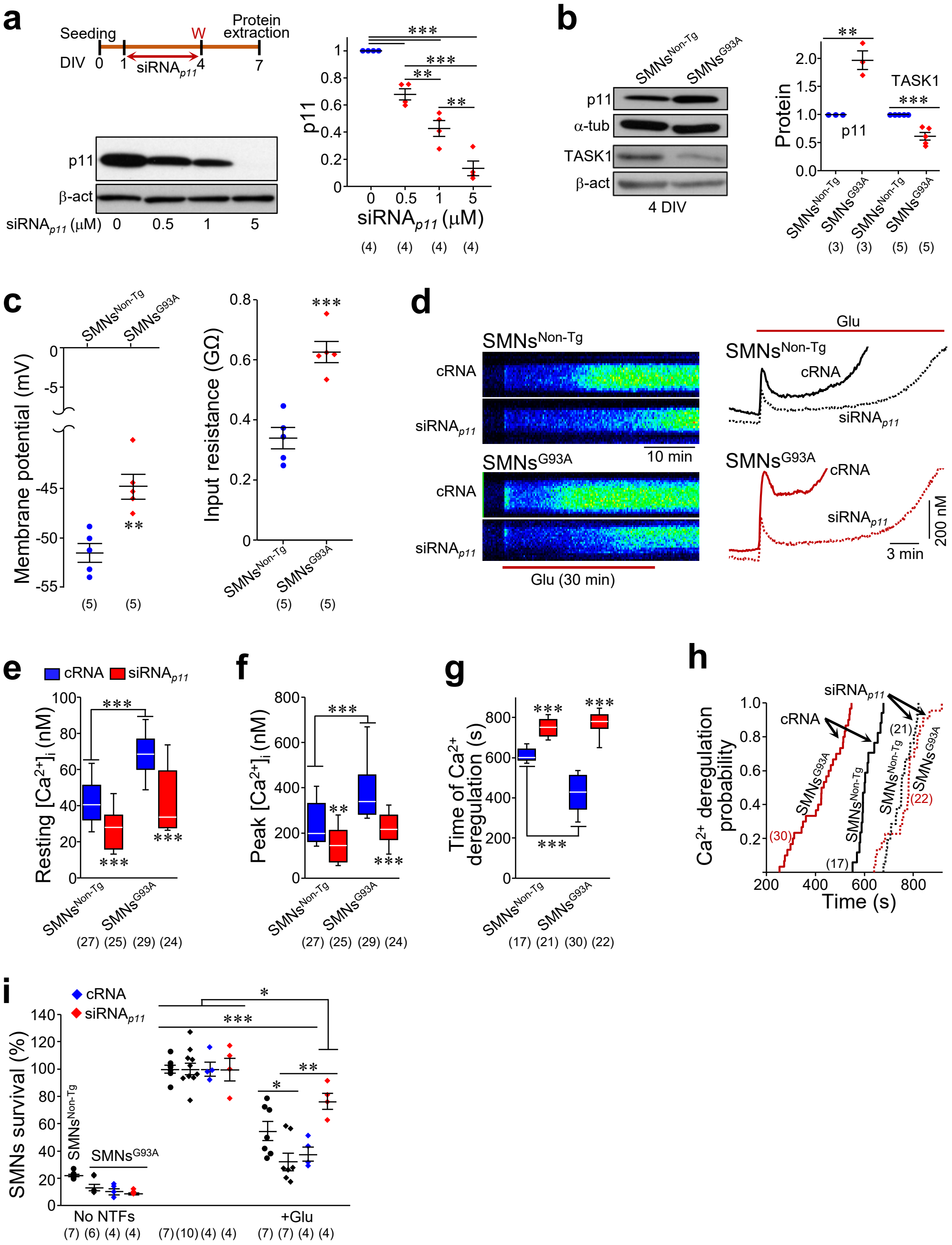

Supplementary Figure 5. p11 downregulation attenuates Ca^{2+} alterations and vulnerability of SMNs^{G93A}. (**a**) Dose-dependent effects of siRNA$_{p11}$ on p11 expression in SMNs^{wt} (see schematic on top for experimental protocol). For each experiment, the value obtained in the absence of siRNA$_{p11}$ was taken as 1. (**b**) Representative immunoblots (left) and histogram (right) showing dysregulation of p11 and TASK1 expression in SMNs^{G93A} and SMNs$^{Non\text{-}Tg}$. *gapdh* and β-act/α-tub were the internal controls for *q*RT-PCR and western blotting, respectively. (**c**) Mean Vm and R_N of recorded SMNs^{G93A} and SMNs$^{Non\text{-}Tg}$ (n = 5 SMNs per group). (**d-g**) Representative dimensional displays (**d, left**) and time course of $[Ca^{2+}]_i$ alterations (**d, right**) obtained from indicated SMNs and treatments. Red horizontal lines indicate the Glu (150 μM) exposure interval. Box-plots of resting $[Ca^{2+}]_i$ (**e**), peak $[Ca^{2+}]_i$ (**f**), and time of Ca^{2+} deregulation (**g**) obtained from SMNs^{G93A} and SMNs$^{Non\text{-}tg}$. (**h**) Log-Rank test, Kaplan-Meier curves of Ca^{2+} deregulation for the SMNs^{G93A} and SMNs$^{Non\text{-}Tg}$ pools treated as stated. (**i**) Glu effects on the survival of SMNs$^{Non\text{-}Tg}$ (circles) and SMNs^{G93A} (diamonds) that received the indicated treatments. All experiments were performed in SMNs at 4-5 DIV. Treatments with cRNA or siRNA$_{p11}$ (2 μM) began at 1 DIV. Number of independent samples is in parentheses. Summary data are shown as mean ± s.e.m. *p < 0.05, **p < 0.01, ***p < 0.001; n.s., not significant; by Student t-test (**b,c**), one-way ANOVA with *post hoc* Holm-Sidak method (**a,e-g,i**). Source data are provided as a Source Data file.

Supplementary Figure 6

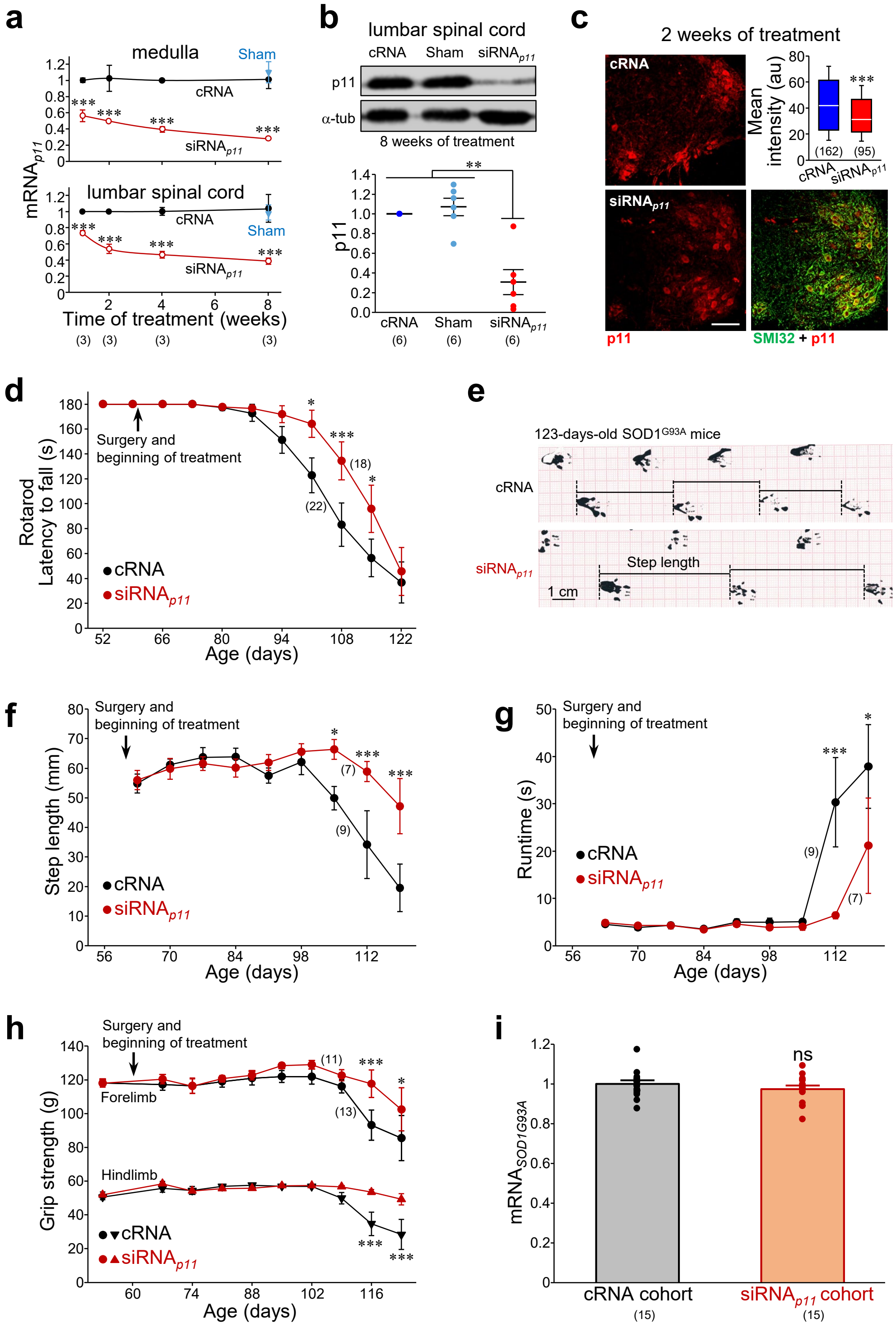

Supplementary Figure 6. siRNA$_{p11}$-induced p11 knockdown delays motor deficits in SOD1^{G93A} mice. (**a**,**b**) Time course of mRNA$_{p11}$ (**a**) and p11 (**b**) expression levels in the medulla oblongata and lumbar spinal cord of adult wild-type mice after oligonucleotide administration. Sham condition is also shown. *gapdh* and α-tub were the internal controls for *q*RT-PCR and for western blotting, respectively. (**c**) Confocal images and mean intensity (arbitrary units, au) of p11 immunolabelling in SMI32-identified lumbar motoneuron pools of adult wild-type mice receiving intracerebroventricular injections of cRNA or siRNA$_{p11}$ for two weeks. Merge image for SMI32 and p11 immunostaining is only shown for the siRNA$_{p11}$ condition. Scale bar, 100 µm. (**d**) Rotarod test was performed to evaluate the time course of deficits in motor coordination, strength and balance, as well as to construct the cumulative probability curve of symptoms onset (see Fig. 3j). (**e-g**) Representative examples of hind footprints from two 123-day-old SOD1^{G93A} littermates receiving the indicated treatments (**e**). How step length measures were obtained is indicated in the records. siRNA$_{p11}$ improved both step length (**f**) and runtime (**g**, time animals took to run a track of 50 cm) decline compared to cRNA. (**h**) Time course of fore (circles) and hind (triangles) limb grip strength (in **g**) decline from cRNA- or siRNA$_{p11}$-treated SOD1^{G93A} mice. (**i**) *q*RT-PCR of DNA isolated from the tail of a random sampling of SOD1^{G93A} mice included in these series of experiments. Number of independent samples is in parentheses. Summary data are shown as mean ± s.e.m. $*p < 0.05$, $**p < 0.01$, $***p < 0.001$; n.s., not significant; by Student *t*-test (**c**,**i**), one-way (**b**) or two-way (**a**,**d**,**f-h**) ANOVA with *post hoc* Holm-Sidak method. Source data are provided as a Source Data file.

Supplementary Figure 7

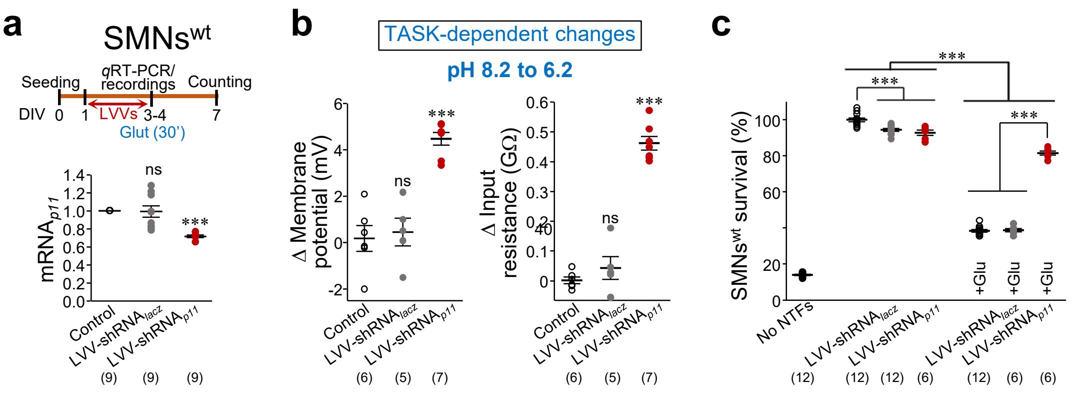

Supplementary Figure 7. LVV-shRNA$_{p11}$ decreases p11, enhances functional expression of TASK-like channels and reduces vulnerability of SMNs^{wt}. Incubation of SMNs^{wt} with LVV-shRNA$_{p11}$ from 1 DIV (see schematic in **a** on top) successfully reduced mRNA$_{p11}$ expression at 3-4 DIV relative to untreated and LVV-shRNA$_{lacz}$ conditions (**a**), robustly increased pH-responsiveness (**b**) and protected SMNs^{wt} against the excitotoxic stimulus Glu (150 µM, 30 min, **c**). Number of independent samples from at least 3 independent experiments is in parentheses. Summary data are shown as mean ± s.e.m. **p* < 0.05, ***p* < 0.01, ****p* < 0.001; n.s., not significant; by one-way ANOVA with *post hoc* Holm-Sidak method (**b**,**c**) or on Ranks with *post hoc* Dunn's method (**a**). Source data are provided as a Source Data file.

Supplementary Figure 8

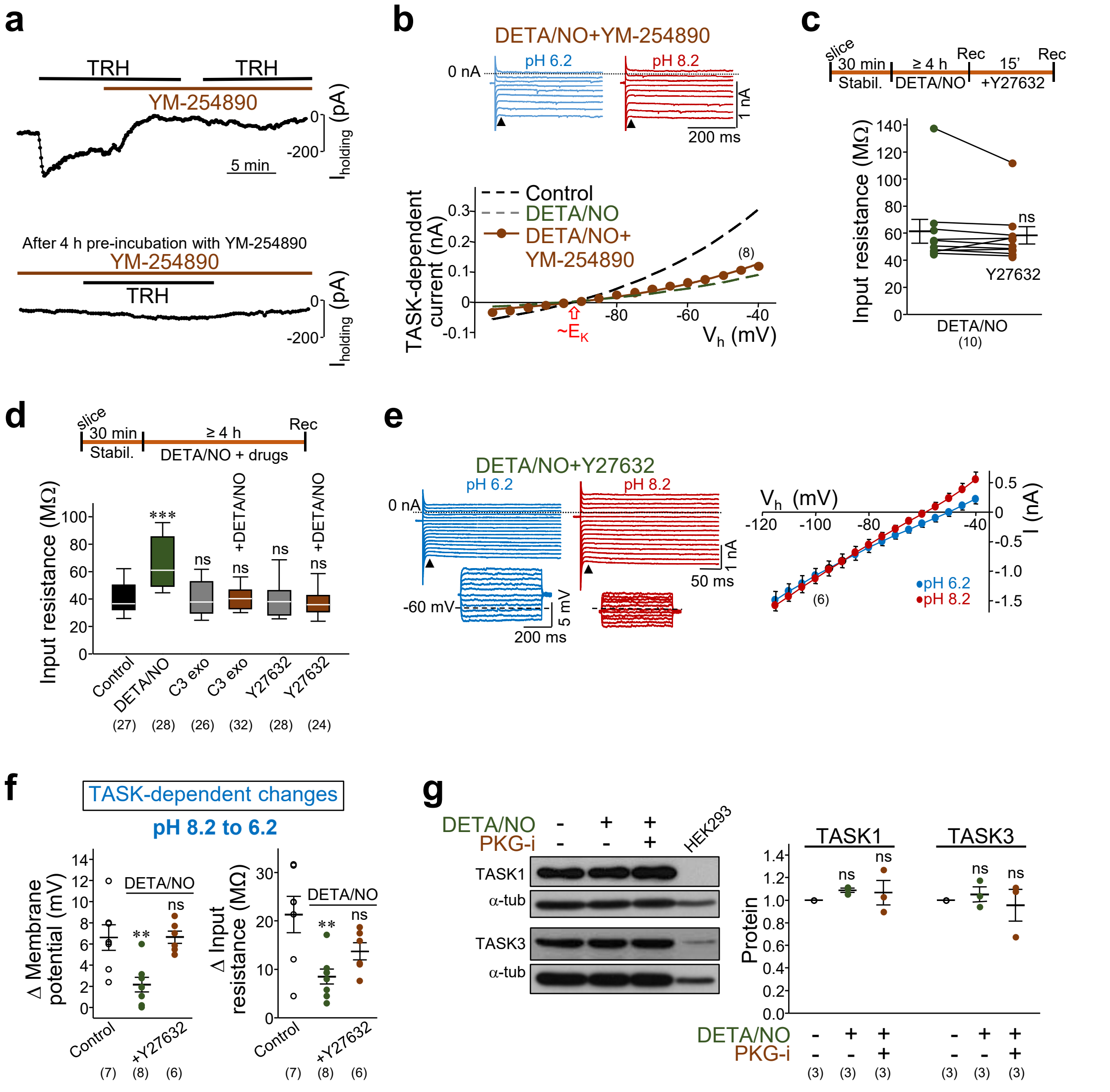

Supplementary Figure 8. Long-term effect of NO on TASK-like currents was independent of Gα_q, short-term ROCK action, and TASK down-expression. (**a**) Changes in the holding current ($I_{holding}$) induced by thyrotropin-releasing hormone (TRH, 10 μM). a Acute or chronic (≥ 4 h) incubation with the Gα_q inhibitor YM-254890 (1 μM) reverted/prevented TRH-induced alterations. (**b**) Current responses to voltage-step commands (range, -115 to -40 mV; 5 mV increments) recorded under indicated pHs from a HMN after chronic incubation (≥ 4 h) with DETA/NO (1 mM) + YM-254890. Alternant current responses have been omitted for clarity. Instantaneous leak currents, measured at the end of the capacitive transient (triangles), were used to construct the averaged I–V relationships (bottom) of the pH-sensitive current component obtained after subtracting mean instantaneous currents at pH 6.2 from those at pH 8.2. As in figure 5h, Goldman–Hodgkin–Katz fits for control and DETA/NO (dashed lines), previously reported[10], are shown here for comparison. (**c**) Plot showing that the effect of chronic incubation with DETA/NO on R_N was unchanged by addition of Y27632 (10 μM, 15 min). Rec indicates when R_N was measured. (**d**) Box-plot of R_N for HMNs recorded after incubation for at least 4 h with the indicated drugs. Exoenzyme C3 (C3 exo) and Y27632 are inhibitors of Rho GTPase and ROCK, respectively. (**e**) Left, as in **b** but for HMNs recorded after chronic incubation with DETA/NO + Y27632. Insets, as in figure 5i from the same motoneuron at the indicated extracellular pHs. Right, averaged data of instantaneous I–V relationships in the control and chronically treated groups in response to extracellular acidification and alkalization. (**f**) Plot represents pH-induced changes (from pH 8.2 to 6.2) in Vm and R_N for each listed treatment group. (**g**) Immunoblots (left) and histogram (right) showing the averaged ratio of TASK1 and TASK3 subunits in the HN of neonatal brainstem slices incubated for 6 h in aCSF alone (control) or supplemented with either DETA/NO (1 mM), or DETA/NO + PKG-i (10 μM). The cell line HEK293 was used as a control. α-tub was the internal loading reference. Number of independent samples is in parentheses. Summary data are shown as mean ± s.e.m. *$p < 0.05$, **$p < 0.01$, ***$p < 0.001$; n.s., not significant; by paired Student *t*-test (**c**), one-way ANOVA with *post hoc* Holm-Sidak method (**d,f**) or on Ranks with *post hoc* Dunn's method (**g**). Source data are provided as a Source Data file.

Supplementary Figure 9

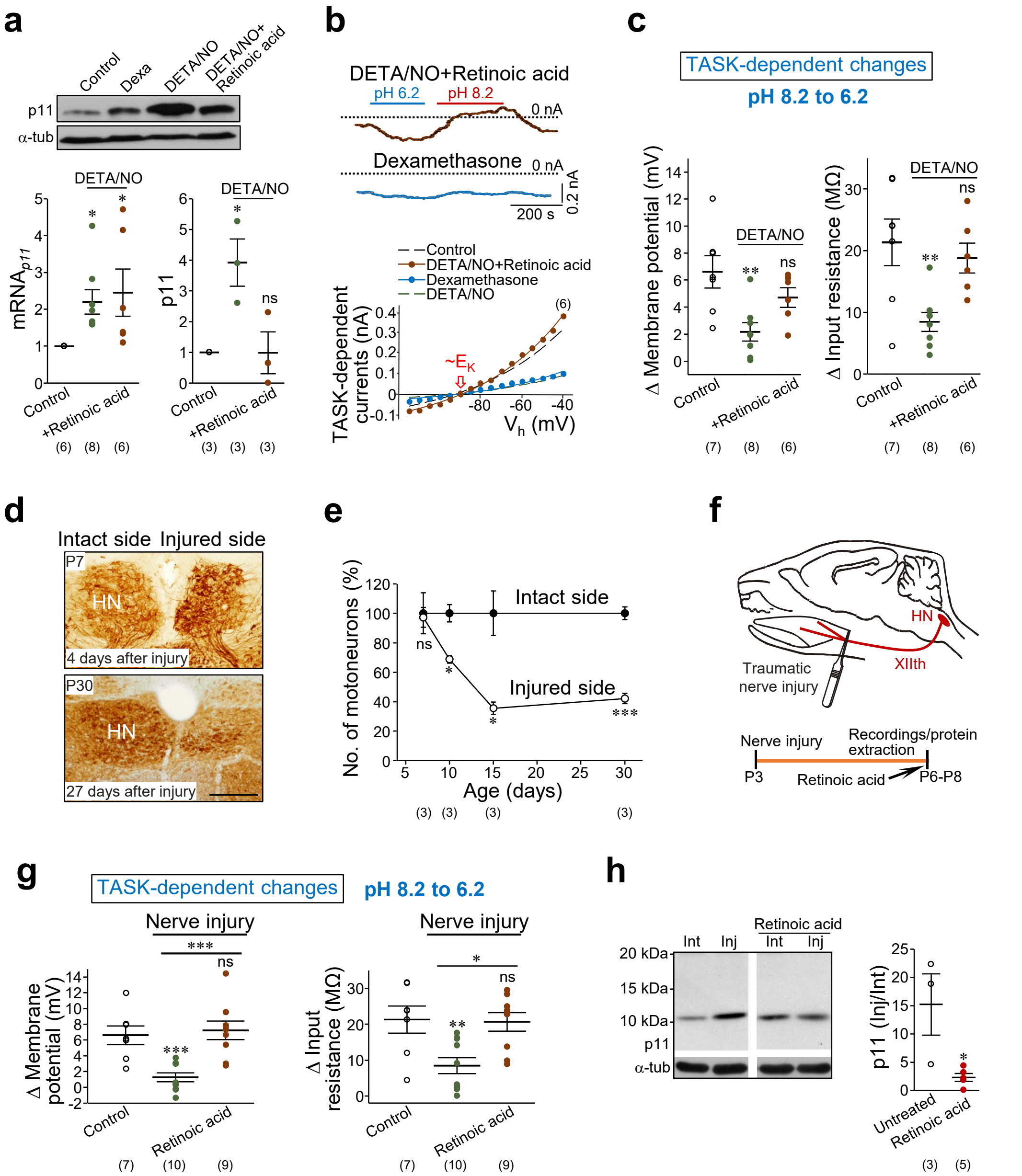

Supplementary Figure 9. Retinoic acid prevents/reverses RNS/nerve injury-induced p11 upregulation and inhibition of TASK-like channels. (**a-c**) Co-incubation with retinoic acid (20 μM. ≥ 4 h) fully avoided both, DETA/NO-induced up-regulation of p11 protein, but not mRNA levels (**a**), and DETA/NO-evoked inhibition of TASK channels in motoneurons (**b,c**). For more details see figure 5h and supplementary figure 8a,b. Goldman–Hodgkin–Katz fits for control and DETA/NO (dashed lines), previously reported[10], are shown here for comparison. Results for dexamethasone (Dexa) have been presented in supplementary figure 1 and they are additionally presented here with comparative purposes. (**d,e**) SMI32-immunolabelled brainstem sections obtained at the level of the HN from two injured animals (**d**) and mean number of HMNs (**e**) in the intact and injured sides at the indicated ages. XIIth nerve crushing was inflicted at P3 (see **f**). Mean number of HMNs in the intact HN was taken as 100% for each time point tested. Scale bar, 300 μm. *n* = 3 rats/group. (**f**) Experimental model. Crushing was inflicted to the XIIth nerve of P3 rats. Pups were untreated and brainstem slices were incubated with retinoic acid (≥ 4 h) before HMN recordings or protein extraction as indicated in the schematic (bottom). (**g**) Plot represents pH-induced changes (from pH 8.2 to 6.2) in Vm and R_N for each listed treatment group. (**h**) Immunoblots for p11 in HN samples obtained from brainstem slices of lesioned animals following incubation with retinoic acid. Plot shows averaged expression of p11 in the injured *versus* intact HN for each experimental condition. Control and/or DETA/NO groups in **c,g** and **h** are the same as for supplementary figure 8f and figure 5g, respectively. *gapdh* and α-tub were internal controls for *q*RT-PCR and western blotting, respectively. Number of independent samples is in parentheses. Summary data are shown as mean ± s.e.m. **p* < 0.05, ***p* < 0.01, ****p* < 0.001; n.s., not significant; Mann-Whitney *U* test (**e,h**), one-way ANOVA with *post hoc* Holm-Sidak method [**a(p11),c,g**] or on Ranks with *post hoc* Dunn's method [**a(mRNA$_{p11}$)**]. Source data are provided as a Source Data file.

Supplementary Figure 10

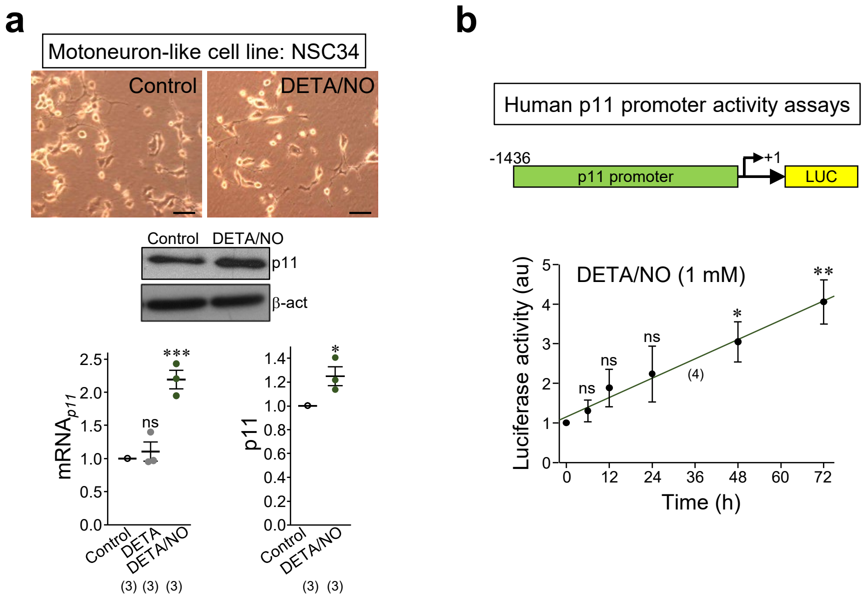

Supplementary Figure 10. RNS increases human p11 promoter activity. (**a**) Validation of the motoneuron-like cell line NSC34 as a model to study NO-mediated induction of p11. Photomicrographs of differentiated cultures (dNSC34) under control condition (untreated) or after exposure to DETA/NO (1 mM) for 48h. Scale bars: 40 µm. DETA/NO treatment for 48 h increased mRNA$_{p11}$ and p11 levels in dNSC34s. DETA, obtained after 48h of DETA/NO (1 mM) in aqueous solution, was taken as an additional control. *gapdh* and β-act were the internal controls housekeeping for *q*RT-PCR and western blotting, respectively. (**b**) Luciferase activity measured in dNSC34 cells co-transfected with the reporter construct pGL4.10-p11-Fir and the normalizer vector pGL4.74-TK-Ren. Plot illustrates the effect of DETA/NO (1 mM) added to the culture medium for several time intervals on promoter activity. Fir-luciferase activity was always normalized relative to Ren-luciferase activity. Luciferase activity is expressed in arbitrary units (a.u.). Number of independent samples is in parentheses. Summary data are shown as mean ± s.e.m. *$p < 0.05$, **$p < 0.01$, ***$p < 0.001$; n.s., not significant; by Student *t*-test [**a(p11)**] or one-way ANOVA with *post hoc* Holm-Sidak method [**a(mRNA$_{p11}$),b**]. Source data are provided as a Source Data file.

Supplementary Figure 11

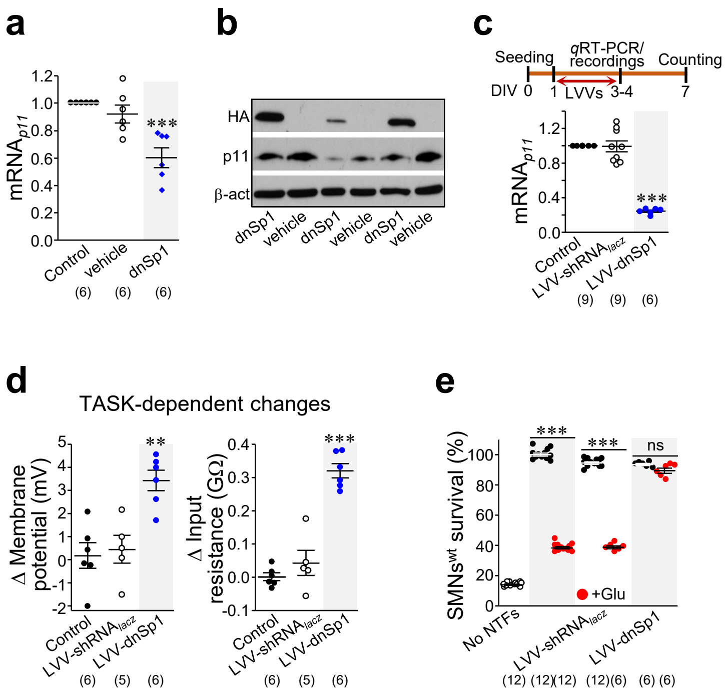

Supplementary Figure 11. A dominant negative of Sp1 (dnSp1) down-regulates p11, promotes functional expression of TASK-like channels and protects motoneurons against an excitotoxic insult. (**a**,**b**) Effects of transient transfection of a plasmid, directing the expression of a dnSp1, on $mRNA_{p11}$ and p11 (**b**) levels in dNSC34s. Three independent experiments were processed in the same immunoblot. Hemagglutinin (HA) expression was an additional marker for transfection efficacy. *gapdh* and β-act were the internal controls for *q*RT-PCR and western blotting, respectively. (**c-e**) Effects of LVV-dnSp1 on $mRNA_{p11}$ levels (**c**, see schematic for experimental design), on pH-induced changes (from pH 8.2 to 6.2) in Vm and R_N (**d**) of $SMNs^{wt}$, and on motoneuron vulnerability to Glu (**e**). $LVV\text{-}shRNA_{lacz}$-treated $SMNs^{wt}$ were used as an additional control condition. Number of independent samples is in parentheses. Summary data are shown as mean ± s.e.m. $^{*}p < 0.05$, $^{**}p < 0.01$, $^{***}p < 0.001$; n.s., not significant; by Student *t*-test (**e**), or one-way ANOVA with *post hoc* Holm-Sidak method (**a**,**c**,**d**). Source data are provided as a Source Data file.

Supplementary Figure 12

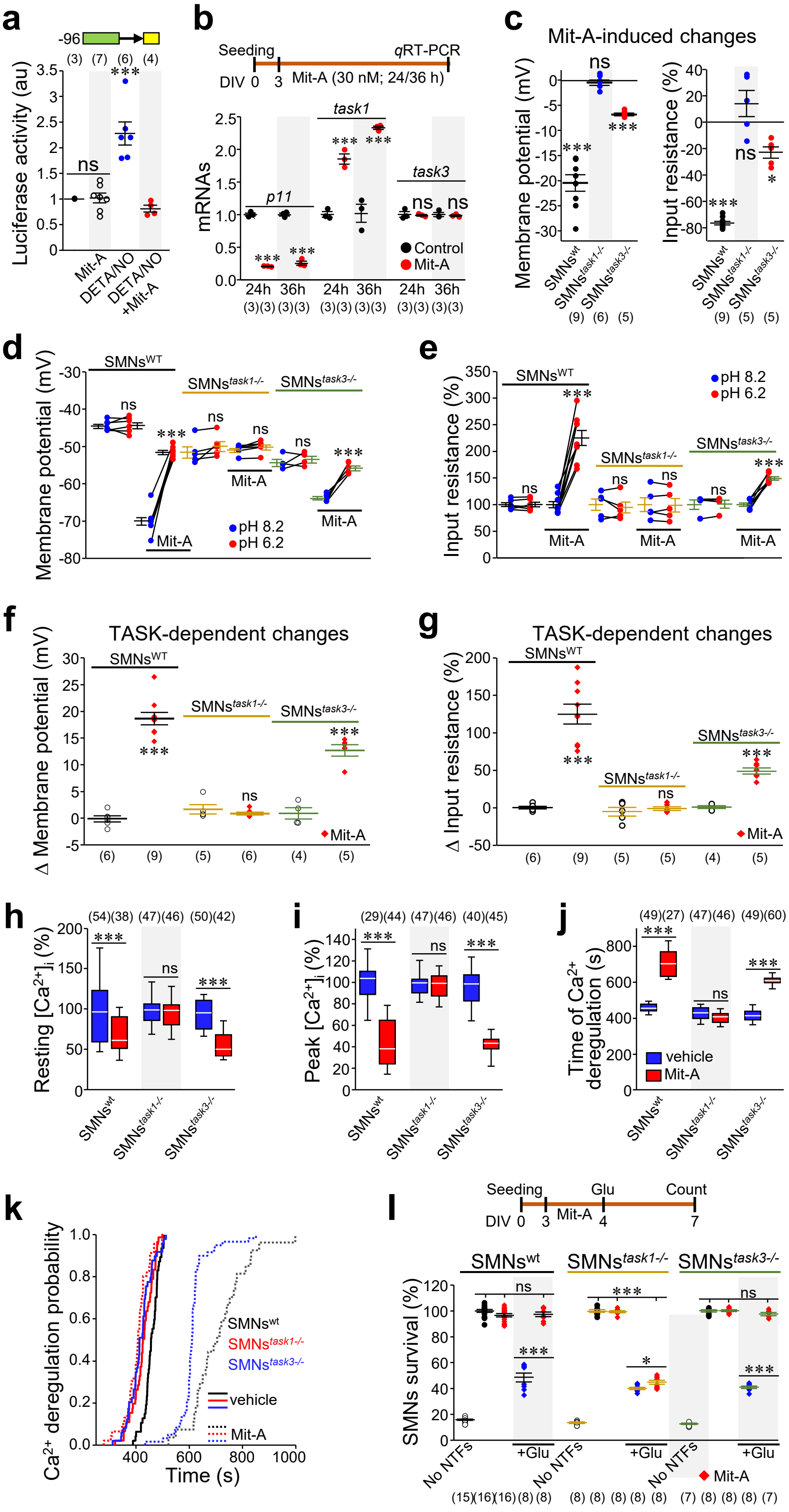

Supplementary Figure 12. Mit-A promotes functional expression of TASK-like channels and attenuates Ca^{2+} alterations via TASK1 subunits. (**a**) Mit-A (30 nM, 48 h) fully avoided DETA/NO-induced (1 mM, 48 h) stimulation of the minimal NO-responsive segment (-96 to +89) of the human p11 promoter. (**b**) mRNA levels for *p11*, *task1*, and *task3* in SMNs^{wt} treated with Mit-A for the stated times (see schematic on top). (**c**) Changes induced by Mit-A on Vm (mV) and R_N (in percent) for the indicated SMN groups relative to those obtained from vehicle-treated pools. (**d-g**) Effects of extracellular pH variation (from pH 8.2 to pH 6.2) on Vm (**d,f**) and R_N (**e,g**) measured in the indicated SMNs pools, that received either vehicle or Mit-A. Individual values (Vm, mV; R_N, in percent) of both parameters for each recorded motoneuron at both pHs are presented in **d** and **e**. The pH-induced changes of Vm (mV) and R_N (in percent) for each analyzed SMN are displayed in **f** and **g**. (**h-j**) Box-plots of resting $[Ca^{2+}]_i$ (**h**), peak $[Ca^{2+}]_i$ (**i**), and time of Ca^{2+} deregulation (**j**) obtained for the specified groups of SMNs treated as indicated in **d-g**. (**k**) Log-Rank test, Kaplan-Meier analysis reported that Mit-A (dotted lines) delayed Ca^{2+} deregulation in SMNs^{wt} and SMNs$^{task3-/-}$ ($p < 0.001$), but not SMNs$^{task1-/-}$ ($p = 0.137$) relative to that in the vehicle condition. Experiments in **c-k** were performed in SMNs at 3-4 DIV. (**l**) Effects of Mit-A on survival of the indicated SMN pools exposed to Glu (30 min, 150 μM). Schematic on top represents the experimental protocol. Number of independent samples is in parentheses. Summary data are shown as mean ± s.e.m. *$p < 0.05$, **$p < 0.01$, ***$p < 0.001$; n.s., not significant; by paired (**d,e**) or unpaired (**b,c,f-j**) Student *t*-test or one-way ANOVA with *post hoc* Holm-Sidak method (**a,l**). Source data are provided as a Source Data file.

Supplementary Figure 13

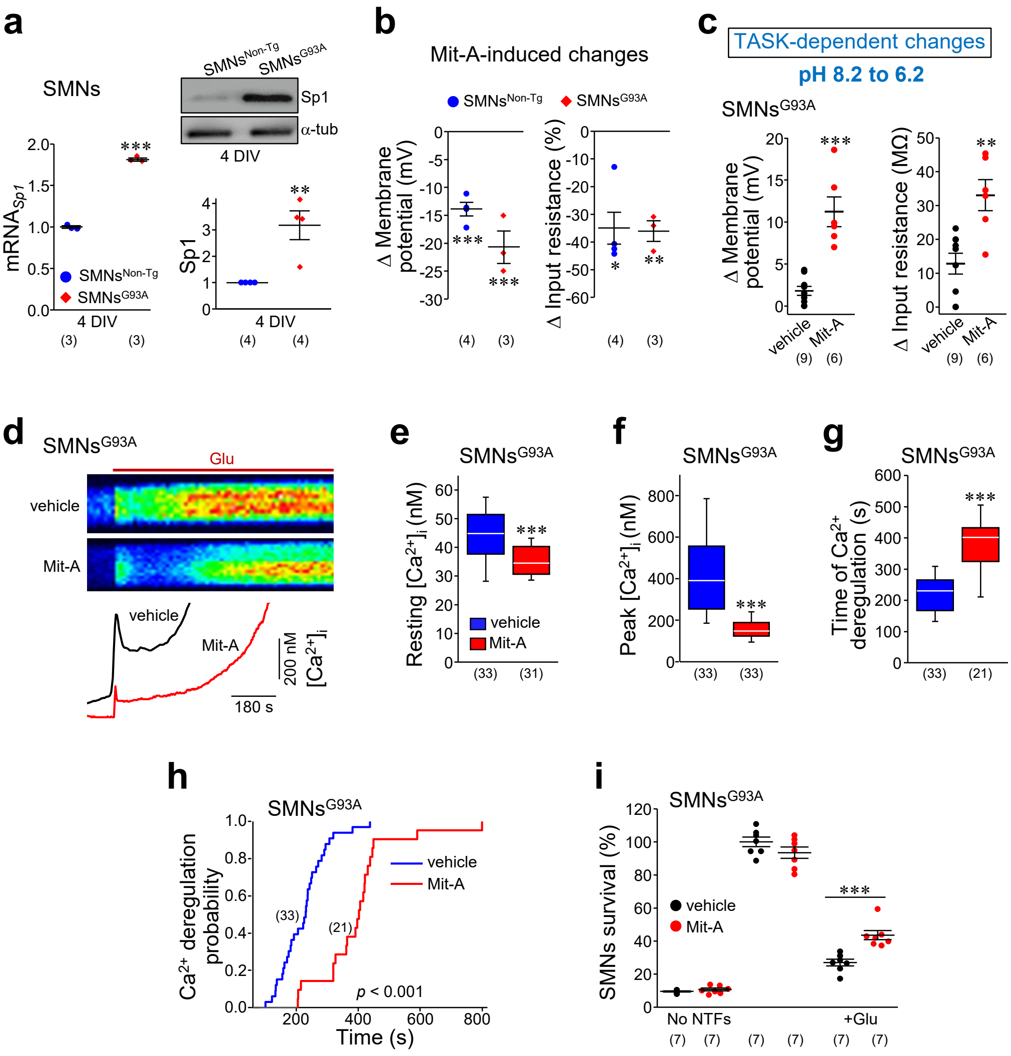

Supplementary Figure 13. Mit-A lowers IME, promotes functional expression of TASK-like channels, attenuates Ca^{2+} disturbance, and reduces vulnerability of SMNs^{G93A}. (**a**) mRNA (left) and protein (right) levels for Sp1 in SMNs^{G93A} and SMNs^{Non-Tg} at 4 DIV. *gapdh* and α-tub were the internal controls for *q*RT-PCR and western blotting, respectively. (**b**) Changes induced by Mit-A on Vm (in mV) and R_N (in percent) for SMNs^{Non-Tg} and SMNs^{G93A} pools relative to those obtained from vehicle-treated pools. (**c**) Effects of Mit-A on pH-induced changes (from pH 8.2 to 6.2) in Vm and R_N of SMNs^{G93A}. (d) Dimensional displays (top) and time course of $[Ca^{2+}]_i$ alterations (bottom) obtained from SMNs^{G93A} that received vehicle or Mit-A. Red horizontal lines indicate Glu (150 μM) exposure interval. (**e-g**) Box-plots of resting $[Ca^{2+}]_i$ (**e**), peak $[Ca^{2+}]_i$ (**f**), and time of Ca^{2+} deregulation (**g**) obtained from SMNs^{G93A} pools. (**h**) Log-Rank test, Kaplan-Meier curves of the time of Ca^{2+} deregulation for SMNs^{G93A} treated as described. (**i**) Effects of Mit-A on survival of the indicated SMN pools exposed to Glu (30 min, 150 μM). Mean number of SMNs in the untreated condition (circles) was taken as 100%. No NTFs, culture medium not supplemented with neurotrophic factors. Experiments in **b-i** were performed in SMNs at 4-5 DIV. Mit-A was added to the culture medium 24-h before. Number of independent samples is in parentheses. Summary data are shown as mean ± s.e.m. *$p < 0.05$, **$p < 0.01$, ***$p < 0.001$; n.s., not significant; by Student *t*-test (**a-c,e-g,i**). Source data are provided as a Source Data file.

Supplementary Figure 14

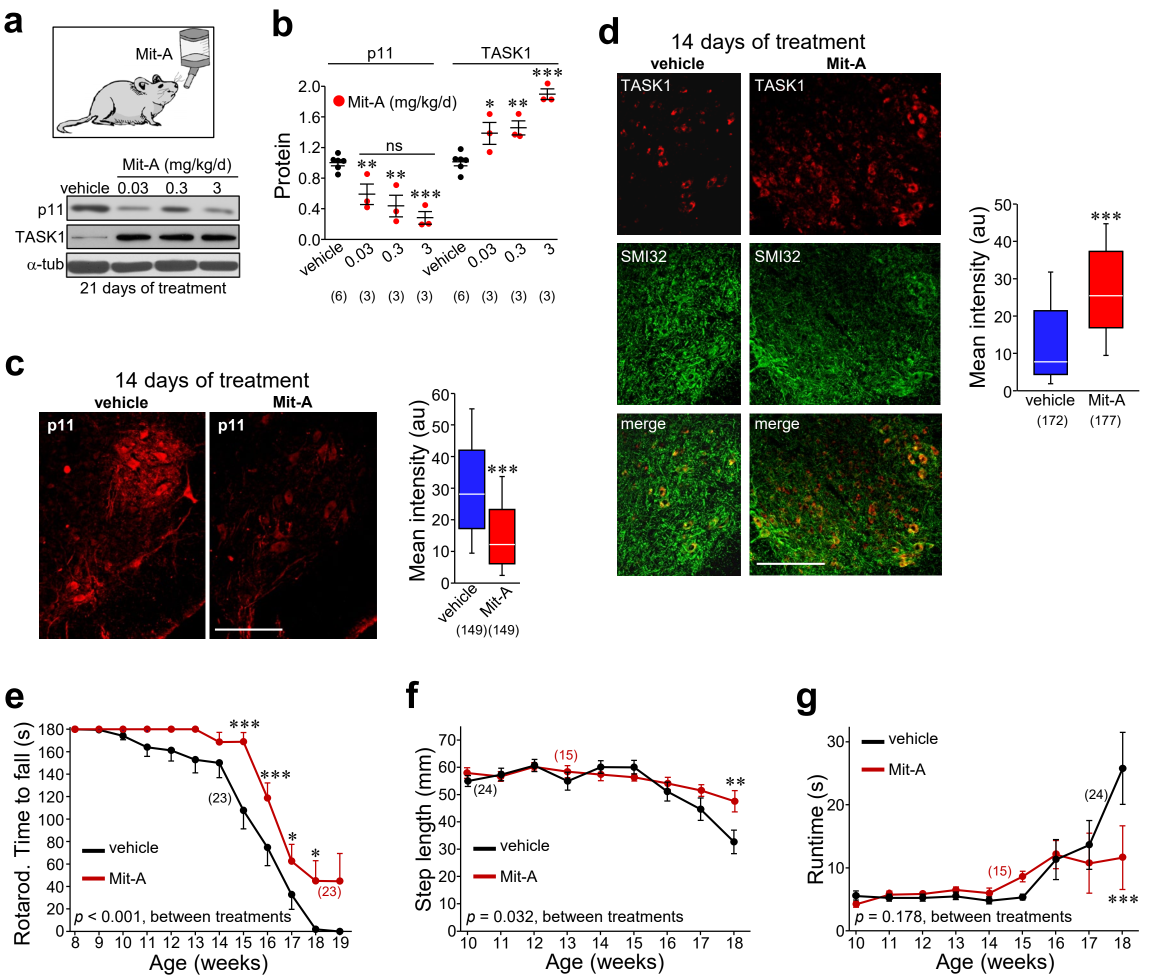

Supplementary Figure 14. Oral administration of Mit-A dysregulates p11/TASK1 expression in lumbar motoneurons and improves motor performance of SOD1^{G93A} mice. (a,b) Effects of oral administration of Mit-A for 3 weeks, at the indicated doses, on p11 and TASK1 expression in the lumbar spinal cord of wild-type adult mice. Mean values obtained from animals treated with vehicle (drinking water supplemented with 1% sucrose) were taken as 1. α-tub was the internal protein loading reference for western blotting. (**c**,**d**) Confocal images of p11 (**c**) and TASK1 (**d**) immunostaining in the ventral horn of the lumbar spinal cord from wild-type mice treated for 2 weeks with vehicle or Mit-A (30 μg/kg/d) in the drinking water. Motoneurons were identified by SMI32 immunolabelling (**d**) in both cases. Box-plots for mean intensity (arbitrary units, au) of p11 (**c**) and TASK1-like (**d**) immunolabelling in SMI32-positive motoneurons. Scale bars, 200 μm. (**e-g**) Latency to fall from rotarod (**e**), step length (**f**), and time required to run along a track of 50 cm (**g**), against the age of SOD1^{G93A} mice receiving the indicated treatments. Mit-A treatment (30 μg/kg/d) began at the pre-symptomatic stage (P30), by drug dissolution in the drinking water, supplemented with 1% sucrose. The vehicle group daily received the same volume of water (1% sucrose). Number of independent samples is in parentheses. Summary data are shown as mean ± s.e.m. $*p < 0.05$, $**p < 0.01$, $***p < 0.001$; n.s., not significant; by Student t-test (**c**,**d**), one-way (b) or two-way (**e-g**) ANOVA with *post hoc* Holm-Sidak method. Two-way ANOVA statistics between treatments are stated on plots. Source data are provided as a Source Data file.

Supplementary Figure 15

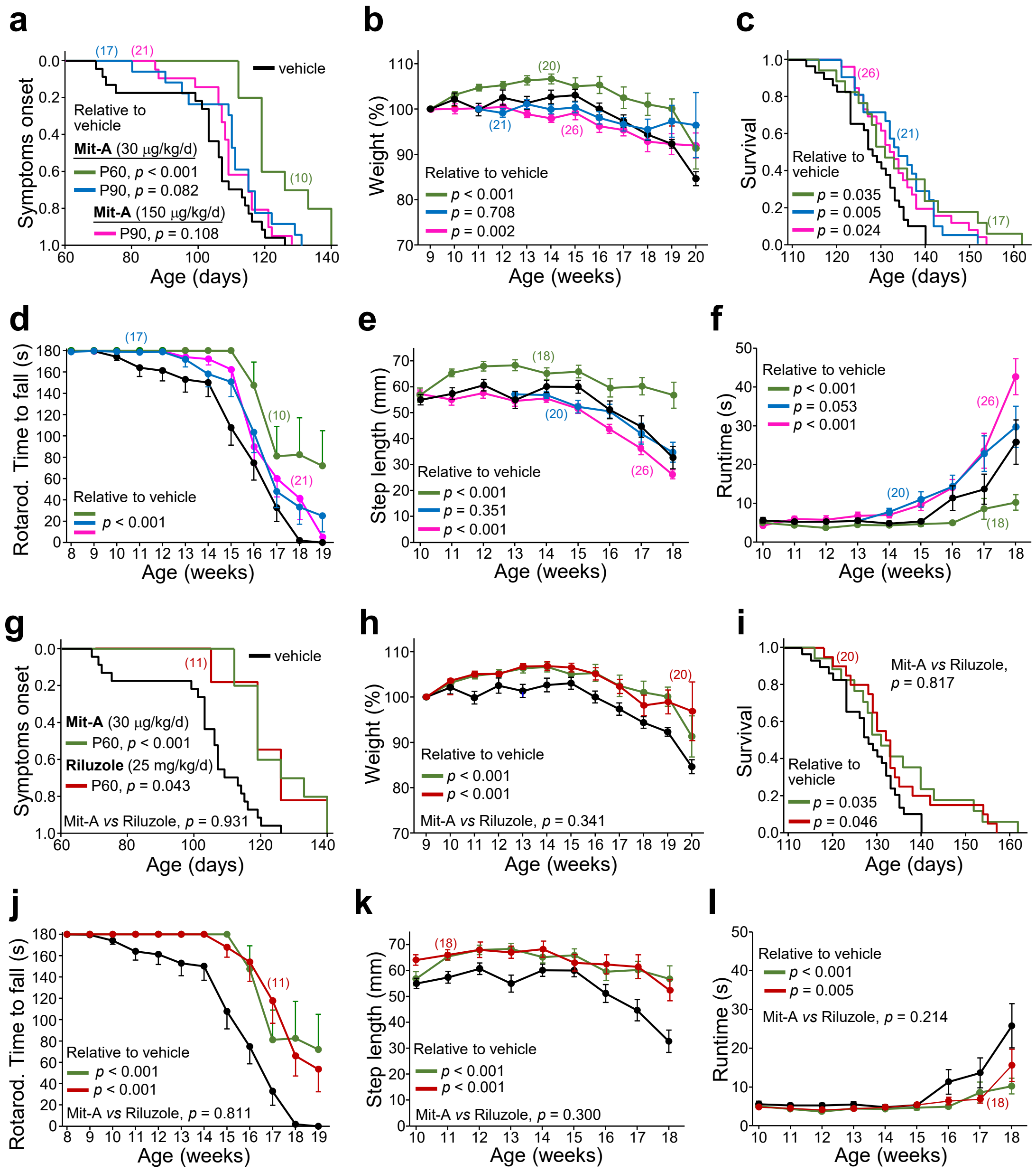

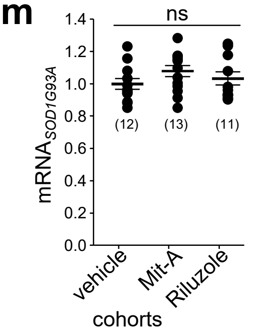

Supplementary Figure 15. Effects of different doses of Mit-A on SOD1^{G93A} mice. Comparison with Riluzole. Cumulative probability curves of symptom onset (**a**,**g**), survival (**c**,**i**), time course of body weight (**b**,**h**), time to fall from rotarod (**d**,**j**), step length (**e**,**k**), and time required to run along a track of 50 cm (**f**,**l**), for SOD1^{G93A} mice receiving the treatments indicated in **a**, for **a-f** and **g**, for **g-l**. (**a-f**) Mit-A treatment (30 μg/kg/d) began at pre-symptomatic (P60) or early symptomatic (P90) stages, by drug dissolution in the drinking water, supplemented with 1% sucrose. A higher dose of Mit-A (150 μg/kg/d) was tested beginning at P90. All animals were treated with vehicle (drinking water supplemented with 1% sucrose) from P30 up to beginning of Mit-A treatment. (**g-l**) Mit-A treatment (30 μg/kg/d) or Riluzole (25 mg/kg/d) began at pre-symptomatic (P60) stage, by drugs dissolution as above. All animals were treated with vehicle (drinking water supplemented with 1% sucrose) from P30 up to beginning of Mit-A/Riluzole treatment. The vehicle and Mit-A groups are the same as for figure 7f-h and supplementary figure 14e-g, and they are shown here for comparison purposes. (**m**) *q*RT-PCR of DNA, isolated from the tail of a random sampling of SOD1^{G93A} mice included in these series of experiments, discarded that differences between treatments were the consequence of different expression degrees of the human transgene. *gapdh* was the housekeeping gene. Number of mice per group and test is indicated in parentheses. Summary data are shown as mean ± s.e.m. n.s., not significant; by one-way ANOVA with *post hoc* Holm-Sidak method (**m**). Statistics outputs determined by Log-Rank test, Kaplan-Meier analysis (**a**,**c**,**g**,**i**) or two-way ANOVA (**b**,**d-f**,**h**,**j-l**) between each Mit-A/Riluzole-treated pools and the vehicle group or between Mit-A and Riluzole are indicated on plots. Source data are provided as a Source Data file.

Supplementary Table 1.- Sequence of primers used for *q*RT-PCR, 5' deletions, and site-directed mutagenesis.

Oligonucleotide primer sequences (5' → 3')

	**For *q*RT-PCR**	
**Gene**	**Forward**	**Reverse**
Human *sod1-G93A*	CCAAGGAGCAGATCATAGG	AGAGCATTGGAGAAGGCAG
p11	CAAAGGAGGACCTGAGAGTGCT	TAGAAAGCTCTGGAAGCCCAC
rhoA	CATCCCAGAAAAGTGGACTCCA	CCTTGTGTGCTCATCATTCCG
sp1	TCTCAGGCAGGCACTATCAGCA	TTGTGATGACACCAAGCTGGC
task1	CTACTACGAGCGCTGGACTTTC	CGAAGCTGAAGGCCACATACT
task3	ATCTTGTGGTCCTGCGATTCC	CGCACGGACAGACACTCTTCTT
gapdh	AGAACATCATCCCTGCATCCA	AGATCCACGACGGACACATTG
	**For 5' deletions**	
**Fragment**		
-1436	AGATCTTGAAGAGCTGTCCCCTTGAA	
-968	AGATCTGTCTCACTCTGTCACCCAGG	
-645	GGGGTAAATCCCCTGTTCAACAA	
-348	CGTGCCCCACCCGCAGGACG	
-188	GCGAGGCCTCTGCGAGGCGG	
-157	AGGGCAGGGCGTGGCCGCGCGCCC	
-126	GGGGGAGTCGGGGGCAGGAAGAG	
-96	AGACAGGGCTGGGGGAGCGCCCT	
-70	GAGCGCCCGCCAGGCTCCTC	
	**For site-directed mutagenesis**	
**Mutation (Mut)**		
Wild-type	AGACAGGGCTGGGGGAGCGCCCTGC	
1	AGACAGGGCTG*ttt*GAGCGCCCT	
2	AGACA*ttt*CTGGGGGAGCGCCCT	
3	AGACAGGGCTGGGGGAG*t*G*ttt*T	
4	AGACAGGGCTGGGGGAGCGCCCTGCCGAGCGCCCGCCAGGCTCCTC CCGCTCCCGCACCGCCTCCCTCTAC*tt*AC*tt*GCCGCACGTA	
5	AGACAGGGCTGG*ttt*AG*t*G*ttt*TGC	

SUPPLEMENTARY REFERENCES

1. Mayer, M.L., Westbrook, G.L. & Guthrie, P.B. Voltage-dependent block by Mg2+ of NMDA responses in spinal cord neurones. *Nature* **309**, 261-263 (1984).
2. Lau, A. & Tymianski, M. Glutamate receptors, neurotoxicity and neurodegeneration. *Pflugers Arch* **460**, 525-542 (2010).
