# Supplementary material for: Sp1-regulated expression of p11 contributes to motor neuron degeneration by membrane insertion of TASK1
Source: Nat Commun. 2019 Aug 22;10:3784. doi: 10.1038/s41467-019-11637-4 (PMC6706379; doi:10.1038/s41467-019-11637-4)
Supplement: Supplementary file 3 — Reporting Summary [file 41467_2019_11637_MOESM3_ESM.pdf]

nature research

Corresponding author(s): Moreno-López, Bernardo

Reporting Summary

Nature Research wishes to improve the reproducibility of the work that we publish. This form provides structure for consistency and transparency in reporting. For further information on Nature Research policies, see Authors & Referees and the Editorial Policy Checklist.

Statistical parameters

When statistical analyses are reported, confirm that the following items are present in the relevant location (e.g. figure legend, table legend, main text, or Methods section).

n/a	Confirmed	
☐	☒	The exact sample size (n) for each experimental group/condition, given as a discrete number and unit of measurement
☐	☒	An indication of whether measurements were taken from distinct samples or whether the same sample was measured repeatedly
☐	☒	The statistical test(s) used AND whether they are one- or two-sided<br>*Only common tests should be described solely by name; describe more complex techniques in the Methods section.*
☐	☒	A description of all covariates tested
☐	☒	A description of any assumptions or corrections, such as tests of normality and adjustment for multiple comparisons
☐	☒	A full description of the statistics including central tendency (e.g. means) or other basic estimates (e.g. regression coefficient) AND variation (e.g. standard deviation) or associated estimates of uncertainty (e.g. confidence intervals)
☐	☒	For null hypothesis testing, the test statistic (e.g. F, t, r) with confidence intervals, effect sizes, degrees of freedom and P value noted<br>*Give P values as exact values whenever suitable.*
☒	☐	For Bayesian analysis, information on the choice of priors and Markov chain Monte Carlo settings
☒	☐	For hierarchical and complex designs, identification of the appropriate level for tests and full reporting of outcomes
☒	☐	Estimates of effect sizes (e.g. Cohen's d, Pearson's r), indicating how they were calculated
☐	☒	Clearly defined error bars<br>*State explicitly what error bars represent (e.g. SD, SE, CI)*

Our web collection on statistics for biologists may be useful.

Software and code

Policy information about availability of computer code

Data collection	pCLAMP 9.2 software (Molecular Devices, Foster City, CA)<br>MiniopticonTM System (BIO-RAD)<br>Olympus FV1000-MPE<br>Personal Molecular Imager™ (PMI™) System, Biorad<br>Rotarod LE8500, Panlab SA<br>Grip Strength Meter, BIOSEB
Data analysis	Clampfit, Molecular Devices, Version 10.5<br>ImageJ 1.48v<br>SigmaPlot (Systat Software, Inc.)

For manuscripts utilizing custom algorithms or software that are central to the research but not yet described in published literature, software must be made available to editors/reviewers upon request. We strongly encourage code deposition in a community repository (e.g. GitHub). See the Nature Research guidelines for submitting code & software for further information.

Data

Policy information about availability of data

All manuscripts must include a data availability statement. This statement should provide the following information, where applicable:

- Accession codes, unique identifiers, or web links for publicly available datasets
- A list of figures that have associated raw data
- A description of any restrictions on data availability

The data that support the findings of this study are available from the corresponding author upon reasonable request.

Field-specific reporting

Please select the best fit for your research. If you are not sure, read the appropriate sections before making your selection.

☒ Life sciences ☐ Behavioural & social sciences ☐ Ecological, evolutionary & environmental sciences

For a reference copy of the document with all sections, see nature.com/authors/policies/ReportingSummary-flat.pdf

Life sciences study design

All studies must disclose on these points even when the disclosure is negative.

Sample size	Sample sizes where chosen in accordance with previous publications and are in line with those generally employed in the field. Sample sizes are considered to be sufficient based on solid statistical results between groups.
Data exclusions	No data were excluded from the analyses.
Replication	Attempts at replication were successful. The number of replicates for each experiment is presented in the figure legends or in the method section.
Randomization	Animals were randomly assigned to the different experimental groups.
Blinding	Investigators were blinded to treatments.

Reporting for specific materials, systems and methods

Materials & experimental systems

n/a	Involved in the study	
☒	☐	Unique biological materials
☐	☒	Antibodies
☐	☒	Eukaryotic cell lines
☒	☐	Palaeontology
☐	☒	Animals and other organisms
☒	☐	Human research participants

Methods

n/a	Involved in the study	
☒	☐	ChIP-seq
☒	☐	Flow cytometry
☒	☐	MRI-based neuroimaging

Antibodies

Antibodies used

pATF-1 (Santa Cruz Biotechnology, Cat# sc-7978, RRID:AB_2086020)
p11 (R&D systems, Cat# AF2377, RRID:AB_2183469)
RhoA (Santa Cruz Biotechnology, Cat# sc-418, RRID:AB_628218)
beta-actin (Sigma-Aldrich, Cat# A5441, RRID:AB_476744)
alpha-tubulin (Sigma-Aldrich, Cat# T9026, RRID:AB_477593)
Sp1 (Abcam, Cat# ab124804, RRID:AB_10974611)
TASK1 (Alomone Labs, Cat# APC-024, RRID:AB_2040132)
TASK1 (Sigma-Aldrich, Cat# P0981, RRID:AB_260876)
TASK3 (Alomone labs, Cat# APC-044, RRID:AB_2039953)

Validation

Validation of primary antibodies are stated in the manufacture's website.

Eukaryotic cell lines

Policy information about cell lines

Cell line source(s)	The Mouse Motor Neuron-Like Hybrid Cell Line (NSC-34) was purchased from CELLutions Biosystems Inc, Toronto, Canada Product code: CLU140
Authentication	NSC-34 cell line was autheticated by CELLutions Biosystems Inc.
Mycoplasma contamination	The cell line was not re-tested for mycoplasma contamination since purchase
Commonly misidentified lines (See ICLAC register)	n/a

Animals and other organisms

Policy information about studies involving animals; ARRIVE guidelines recommended for reporting animal research

Laboratory animals	Neonatal (P3-P9) wistar rats of either sex, young (1-2 months-old) and adult (> 2-months-old) male mice, either CD1, SOD1G93A (Jackson Laboratory, Bar Harbor, ME, USA) and Non-Tg littermates; and, CD1, C57BL/6J, task1-/-, task3-/- and SOD1G93A pregnant mice (12.5 days gestation).
Wild animals	The study did not involve wild animals.
Field-collected samples	This study did not involve field-collected samples
